# Silver-Catalyzed Aqueous Electrochemical Valorization of Soda Lignin into Aliphatics and Phenolics

**DOI:** 10.3390/polym16233325

**Published:** 2024-11-27

**Authors:** Lucie Lindenbeck, Silas Brand, Franka Stallmann, Vanessa Barra, Marcella Frauscher, Björn B. Beele, Adam Slabon, Bruno V. Manzolli Rodrigues

**Affiliations:** 1 Faculty of Mathematics and Natural Sciences, Chair of Inorganic Chemistry, University of Wuppertal, Gaussstraße 20, 42119 Wuppertal, Germany; lindenbeck@uni-wuppertal.de (L.L.); silas.brand@uni-wuppertal.de (S.B.); franka.stallmann@uni-wuppertal.de (F.S.); barra@uni-wuppertal.de (V.B.); beele@uni-wuppertal.de (B.B.B.); 2 AC2T Research GmbH, Viktor Kaplan-Straße 2/c, 2700 Wiener Neustadt, Austria; marcella.frauscher@ac2t.at; 3 Wuppertal Center for Smart Materials & Systems, University of Wuppertal, 42119 Wuppertal, Germany

**Keywords:** electrocatalysis, lignin, silver, depolymerization, dearomatization

## Abstract

Transitioning from crude oil to renewable sources of carbon-based chemicals is critical for advancing sustainable development. Lignin, a wood-derived biomacromolecule, holds great potential as a renewable feedstock, but efficient depolymerization and dearomatization methods are required to fully unlock its potential. In this investigation, we present a silver-catalyzed aqueous electrocatalytic method for the selective depolymerization and partial dearomatization of soda lignin under mild, ambient conditions. Utilizing a water/sodium carbonate solvent system and a silver electrode to mediate the electrochemical reduction, we achieved significant lignin depolymerization over reaction times ranging from 5 to 20 h. Analysis by nuclear magnetic resonance (NMR) and high-resolution mass spectrometry (HRMS) revealed sodium levulinate, sodium acetate, and sodium formate as the main aliphatic products, alongside various aromatic species in the depolymerized lignin products (DL). This selective conversion of lignin into both valuable aromatic compounds and reactive aliphatic intermediates offers promising opportunities for further synthesis of a wide range of organic chemicals, contributing to the development of a more sustainable and circular economy.

## 1. Introduction

The quest for sustainable and renewable sources of organic chemicals has directed significant research attention toward lignin, an abundant macromolecule found in the cell walls of plants. This complex polyphenol is one of the most abundant natural macromolecules, constituting approximately 15–30% of the biomass in vascular plants. Despite its potential as a renewable source of aromatic compounds, its complex and intricate structure presents a major challenge in its depolymerization and subsequent conversion into valuable chemicals [1,2]. Unlike cellulose or hemicellulose, lignin has a highly crosslinked network, which hinders its breakdown using conventional thermal or chemical processes. These challenges have historically limited lignin’s industrial application to low-value uses such as combustion for energy production in pulp mills, but advances in catalysis are now finding new pathways for its valorization [1,2,3,4].

Traditionally considered a waste product in the pulp and paper industry, lignin holds vast potential as a feedstock for valuable aromatic compounds [1,2,5]. In this context, electrocatalysis has emerged as a promising approach for lignin valorization, most specifically its depolymerization, offering significant advantages over traditional methods [3,4,5,6,7]. Conventional methods for lignin depolymerization, such as pyrolysis and hydrogenolysis, operate under extreme conditions of temperature and pressure, which often lead to a wide distribution of products with limited selectivity. As a result, there is growing interest in developing milder, more selective processes, such as Soxhlet extraction and reflux [8], and planetary ball milling [9], which also offer the potential to produce more desirable products with targeted compositions. Similarly, electrocatalysis is another technique that enables lignin depolymerization under milder conditions [4,5], with the potential for greater control over reaction pathways. This method minimizes energy input and reduces the likelihood of side reactions that lead to high-molecular-weight byproducts, enabling more selective conversion of lignin into smaller, more valuable molecules [1,2,5,10].

One of the key benefits of electrocatalysis is its ability to operate at lower temperatures and pressures compared to conventional thermal or chemical processes, which often require harsh conditions and can lead to unwanted by-products [3]. The successful valorization of lignin would represent a dual opportunity: reducing reliance on fossil-based feedstocks for chemical production while also creating economic value from what is currently considered a low-value byproduct of the pulp and paper industry. Converting lignin into high-value chemicals aligns with the principles of a circular bioeconomy, addressing the need for sustainable and renewable sources of carbon-based materials [7]. Additionally, electrocatalytic methods can be finely tuned by adjusting parameters, allowing for greater control over the reaction pathways and product selectivity. The use of renewable electricity further enhances the sustainability of this approach, reducing reliance on fossil fuels and minimizing the environmental impact. Consequently, electrocatalysis holds great potential for transforming lignin into a range of useful chemicals and fuels in a more efficient and eco-friendly manner [3,7].

In a recent study, we demonstrated the efficacy of carbon as an electrocatalyst in achieving selective depolymerization and full dearomatization of lignin in aqueous medium under reductive conditions, leading to valuable aliphatic compounds instead of aromatics [4]. Through a systematic investigation, a current of −175 mA and a reaction time of 20 h were established as the optimal parameters for soda lignin depolymerization/dearomatization, when carbon was used as an electrocatalyst. This previous work not only highlighted the feasibility of utilizing lignin as a resource for aliphatic organic chemicals but also set the stage for exploring alternative electrocatalysts to further enhance the process. Building on this foundation, we have turned our attention to silver as a potential electrocatalyst. This transition metal has garnered significant attention as an electrocatalyst due to its exceptional electrical conductivity, catalytic efficiency, and relatively low cost compared to other noble metals like platinum [11,12,13,14,15,16]. Silver stands out among electrocatalysts due to its unique ability to selectively activate oxygen-containing functional groups while maintaining stability across a variety of electrochemical conditions. Its ability to engage in both reductive and oxidative transformations makes it particularly suited for biomass conversions where complex multifunctional molecules, like lignin, are involved [12]. Compared to other materials, silver’s interaction with aromatic groups in lignin may facilitate not only depolymerization but also selective partial dearomatization, a crucial step in producing valuable aliphatic intermediates. Furthermore, its relatively low overpotential for electrochemical reductions suggests it can drive these reactions with greater energy efficiency compared to other catalysts like carbon or copper [9,10]. Mechanistic studies on silver-containing catalysts for lignin depolymerization, however, are scarce and limited, for example, to silver-copper oxide [17] or Ag_2_S@CdS redox catalysts used in the photodegradation of lignin [18]. Regardless, its unique surface properties facilitate various electrochemical reactions, making it a versatile material for applications in fuel cells [19,20,21], batteries [22,23], and the reduction of carbon dioxide to valuable chemicals [22,24]. The high surface area of nanostructured silver enhances its catalytic performance, providing a larger number of active sites for reactions [13,14].

**Scheme 1:**
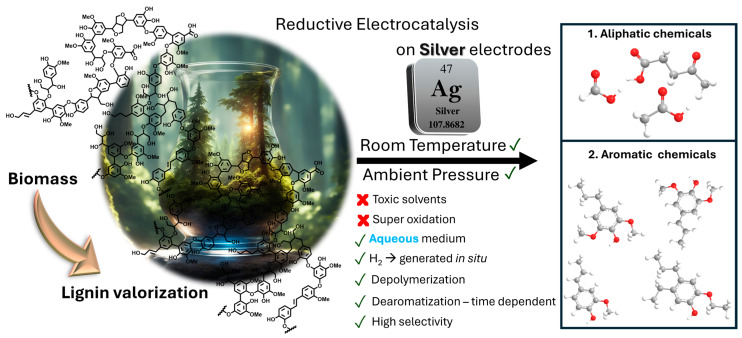
Schematic representation of the silver-catalyzed electrocatalytic depolymerization and partial dearomatization of soda lignin in an aqueous sodium carbonate medium. The process involves the application of a constant current (−175 mA), driving the electrochemical reduction of lignin at ambient temperature and pressure. The selective cleavage of lignin results in the formation of both aliphatic compounds (e.g., sodium levulinate, sodium formate, and sodium acetate) and aromatic species, which are extracted using ethanol as a solvent. The mechanism highlights the role of silver as an electrocatalyst in enhancing product distribution and selectivity compared to previous systems.

In this follow-up investigation, we investigate the use of silver as an electrocatalyst in the reductive depolymerization of lignin toward organic chemicals, both aliphatics and phenolics (Scheme 1). Our objectives centered on the comparison between the performance of silver with that of carbon electrodes [4,25], while assessing the influence of silver on product distribution and setting the grounds to understand the underlying mechanisms driving the enhanced reactivity. By leveraging the catalytic properties of silver, we aimed to achieve higher yields of valuable organic chemicals and further our understanding of the fundamental processes involved in lignin electrocatalysis.

## 2. Materials and Methods

### 2.1. Materials

Soda lignin was obtained from WAT Venture Sp. z o.o., Warsaw, Poland, which was extracted from the hardwood black liquor of a sulfur-free pulp production in a pilot plant. A silver wire (0.5 mm diameter, 99.9% purity) from AlfaAesar (Haverhill, MA, USA) was used as the working electrode. Sodium carbonate (≥99.5%, ACS reagent, Merck (Darmstadt, Germany)), Methanol (MeOH, LC-MS grade, Fisher Scientific (Schwerte, Germany)), and ethanol (puriss. p.a., absolute, ≥99.8% (GC), Sigma Aldrich, St. Louis, MO, USA) were used as received. All aqueous solutions were prepared with ultrapure water, obtained from a Millipore system.

### 2.2. Lignin Depolymerization

All electrochemical reactions were carried out using an ATLAS 1131 Electrochemical Unit & Impedance Analyser (Atlas Sollich, Kartuzy County, Poland). In a standard experiment, soda lignin was initially dissolved at a concentration of 3 g∙L^−1^ in 5 mL of a 1 M aqueous sodium carbonate solution. Subsequently, 45 mL of water was added to this mixture. The electrochemical depolymerization process was conducted with a three-electrode system: a silver wire working electrode, a platinum wire counter electrode, and an Ag/AgCl (saturated KCl) reference electrode. Chronopotentiometry was performed with a constant current of −175 mA for different reaction times (up to 20 h). All experiments were conducted at room temperature and ambient pressure. Post-reaction, water was removed under reduced pressure, and the resulting solid was dried under the same conditions. The dried solid was then suspended in ethanol and stirred vigorously for 1 h. The remaining residue was filtered off, and ethanol was evaporated from the filtrate under reduced pressure, yielding a white solid.

**0 h:** FTIR (Diamond-ATR): ν [cm^−1^] = 3313 (O-H, w), 2961 (C-H, w), 2918 (C-H, w), 2873 (C-H, w), 2850 (C-H, w), 1717 (C=O, m), 1700 (C=O, m), 1652 (m), 1594 (C=C, m), 1443 (C=C, m), 1346 (s), 1258 (C-O, s), 1015 (C-O-C, C-O-H, s), 877 (m), 866 (m), 835 (C-H, s), 798 (CH, s), 668 (s), 400 (m).
**5 h:** FTIR (Diamond-ATR): ν [cm^−1^] = 3566 (O-H, w), 2918 (C-H, w), 2873 (C-H, w), 2851 (C-H, w), 1772 (w), 1733 (C=O, m), 1717 (C=O, m), 1700 (C=O, m), 1652 (m), 1589 (C=C, m), 1521 (C=C, m), 1374 (s), 1349 (s), 1249 (C-O, m), 948 (m), 878 (m), 837 (C-H, s), 767 (m), 700 (C-H, m), 668 (s), 420 (s).
**10 h:** FTIR (Diamond-ATR): ν [cm^−1^] = 3310 (OH, w), 2917 (CH, m), 2850 (CH, m), 1728 (C=O, m), 1595 (C=C, m), 1565 (m), 1347 (C-H, s), 1260 (C-O, s), 1076 (C-O, s), 1049 (s), 982 (s), 833 (C-H, s), 767 (m), 654 (s), 569 (s).
**15 h:** FTIR (Diamond-ATR): ν [cm^−1^] = 3350 (O-H, w), 2954 (C-H, w), 2916 (C-H, w), 2872 (C-H, w), 2850 (C-H, w), 1733 (C=O, m), 1717 (C=O, m), 1700 (C=O, m), 1652 (m), 1635 (m), 1564 (C=C, m), 1456 (C-H, m), 1048 (s), 1012 (C-O-C, C-O-H, s), 833 (C-H, s), 781 (m), 668 (s), 400 (m).
**20 h:** FTIR (Diamond-ATR): ν [cm^−1^] = 3399 (O-H, w), 2962 (C-H, w), 2917 (C-H, w), 1717 (C=O, m), 1700 (C=O, m), 1591 (C=C, m), 1564 (C=C, m), 1441 (C-H, m), 1259 (C-O, s), 1078 (s), 1015 (C-O-C, C-O-H, s), 832 (C-H, s), 798 (C-H, s), 668 (s), 404 (m).

### 2.3. Characterization

**Nuclear Magnetic Resonance Spectroscopy (NMR):** ¹H NMR spectra were obtained using a Bruker Avance III 600 NMR instrument (Bruker Corporation, Billerica, MA, USA) at a temperature of 300 K in deuterium oxide (D_2_O). The following sample probes were employed: a 5 mm broadband inverse probe with automatic frequency determination, a 5 mm QNP probe, and a 5 mm broadband inverse probe. Chemical shifts were referenced against tetramethylsilane (Me_4_Si) for ¹H. Unless otherwise specified, 16 scans were recorded with a 1.0 s delay between each scan for the ¹H NMR spectra.
**Vibrational spectroscopy (FTIR):** Fourier-transform infrared (FTIR) spectra were measured using a Nicolet iS5 spectrometer (Thermo Scientific, Waltham, MA, USA), equipped with an iD5 diamond attenuated total reflection (ATR) unit.
**Direct infusion (DI) ESI-HRMS:** Depolymerized lignin samples were dissolved in methanol, ultrasonicated for 30 min, and centrifuged for 10 min (14,000 rpm). High-resolution mass spectrometry (HRMS) was used as an advanced analytical method to gain the structure information of degradation products of lignin. MS and MSn spectra were obtained using an Orbitrap-IQX high-resolution mass spectrometer (ThermoFisher Scientific, Bremen, Germany), equipped with an ESI source. ESI-MS analyses were carried out in ESI (−) and ESI (+) mode. The solutions were infused into the ESI source via direct infusion (DI) at a rate of 5 µL min^−1^. Typical spray and ion optics for negative mode conditions were the following: source voltage, 3.0 kV; sheath gas flow rate, 8 arb; capillary temperature, 275 °C; capillary voltage, −50 V; tube lens voltage, −130 V. Fragmentation and interpretation were performed based on negative ionization mode; positive ionization mode was used for additional confirmation. Xcalibur version 2.0.7 and Mass Frontier version 8.0 (ThermoFisher Scientific, Bremen, Germany) software were used for data processing and evaluation.

## 3. Results and Discussions

In this work, we extended our investigation toward the electrochemical reductive depolymerization of soda lignin [4,25], by using silver as an electrocatalyst. This process involved dissolving soda lignin in an aqueous sodium carbonate solution, followed by an electrocatalytic reaction aimed at the simultaneous depolymerization and partial dearomatization of lignin to produce valuable aliphatic products. Prior to its utilization as an electrocatalyst, the stability of the electrode was evaluated through cyclic voltammetry (CV) (Figure S1) in a lignin solution. No evidence of electrode oxidation was discerned, thereby indicating that the electrode remains stable under the chosen conditions. The choice of −175 mA as the applied current was based on previous work, where this current was shown to be the optimal one [4]. Following the depolymerization process, the resulting depolymerized lignin (DL) products were extracted through solid–liquid extraction using ethanol as the extractant. Ethanol was carefully chosen due to its specific incompatibility with our electrolyte, sodium carbonate. This selection minimized unwanted interactions between the extractant and the electrolyte, facilitating a cleaner separation of the products. Additionally, ethanol offers several practical benefits for our extraction process. One key advantage is its potential for reuse, allowing for repeated extractions without significant loss of efficacy. Moreover, ethanol is regarded as a safe and environmentally friendly solvent, recognized by CHEM21 for its sustainable profile in chemical processes.

**Figure 1:**
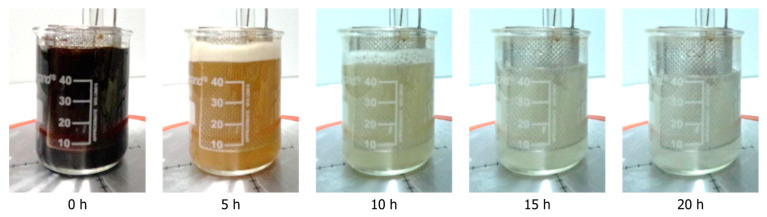
The color of the solution changes during the depolymerization process in a time-dependent manner, becoming clearer as the reaction progresses. This observation serves as an indirect indicator of the extent of depolymerization.

The dissolution of soda lignin in an aqueous sodium carbonate results in the formation of a dark brown solution (Figure 1, 0 h). It is well established that the reaction time and applied current exert a significant influence on the decolorization of the lignin solution during the depolymerization process [4]. In earlier studies, the extent of decolorization was employed as an indirect indicator of depolymerization [4,26]. The decolorization of the lignin solution during the reaction is readily apparent. At the completion of the reaction, a solution that is entirely devoid of color and transparent is observed. Additionally, the volume of the reaction mixture undergoes a reduction over the course of the reaction, which can be attributed to water splitting (E > 1.23 V vs. RHE) [27].

The time-dependent reaction yields (Table 1) showed an increase throughout the depolymerization process, from 5 to 20 h. In this case, the yield represents the fraction of depolymerization products (DL) that are ethanol-soluble, relative to the mass of the starting soda lignin. Notably, in the reference reactions (soda lignin powder and 0 h), products could also be extracted with ethanol, but their yields were significantly lower compared to the reactions where electrocatalysis was employed. After 10 h, a slight decline in yield was observed, which may be attributed to extensive lignin depolymerization resulting in the formation of small volatile products. Since the reaction occurs in an open system, these volatile products may escape, potentially explaining the observed decrease in yield after 10 h.

**Figure 2:**
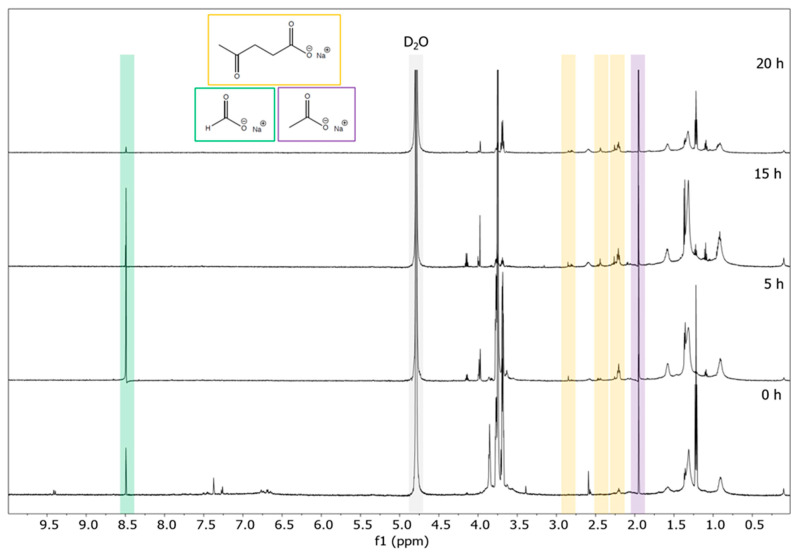
^1^H NMR spectra (D_2_O, 600.13 MHz) of DL for different reaction times: 0 h, 5 h, 15 h, and 20 h. Levulinate (yellow), formate (green), and acetate (purple) could be identified as main aliphatic products.

NMR spectroscopy is established as a non-invasive and fast spectroscopic method for characterizing both lignin structural motifs [28,29,30,31,32] and valuable depolymerization products. [4,5,25] Using ^1^H NMR spectroscopy, we observe a decrease in aromatic product with time as well as the occurrence of characteristic resonances of three main depolymerization products: sodium levulinate, sodium formate, and sodium acetate (Figure 2). In our previous work, in which carbon was used as an electrocatalyst for the depolymerization of soda lignin, sodium 4-hydroxyvalerate was also identified as a major organic aliphatic product [4]. It was assumed that gamma-valerolactone (GVL) was present as an impurity in the starting lignin, as this solvent is often used to extract lignin from wood [33]. Therefore, sodium 4-hydroxyvalerate would result from the alkaline-catalyzed ring-opening of GVL. The absence of this product in the DL products indicates that silver, as an electrocatalyst, may have played a role throughout the reaction, not preserving this aliphatic compound in the final products but instead converting it into smaller fractions. Since no methanol was detected in these experiments, we can confirm that depolymerization does not follow the Kolbe pathway in our catalyst system [34].

**Figure 3:**
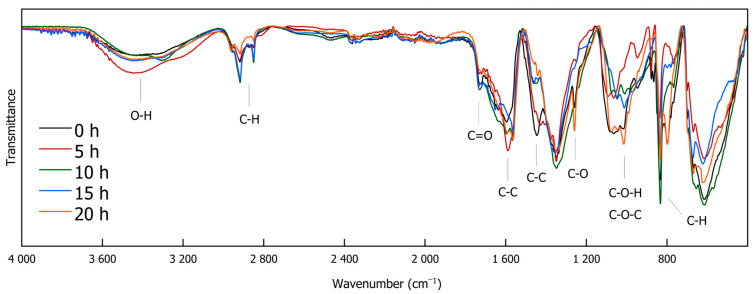
FTIR spectra of DL for different reaction durations (0 h, 5 h, 10 h, 15 h, and 20 h).

**Figure 4:**
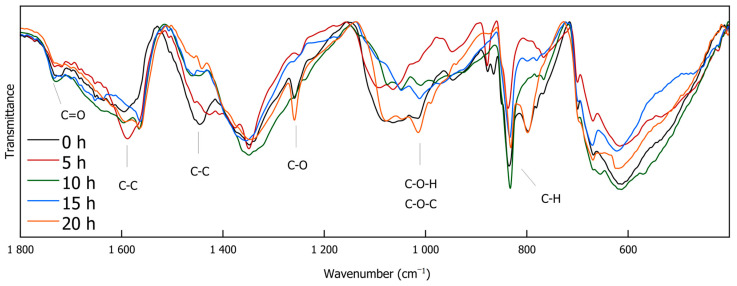
FTIR spectra of DL for different reaction durations zoomed in in the region between 1800 and 400 cm^−1^ (0 h, 5 h, 10 h, 15 h, and 20 h).

IR spectroscopy is a versatile, non-destructive analytical technique that provides information on functional groups within a sample. It is widely used in various scientific fields, such as characterizing modern nanocomposite materials [35], elucidating structural motifs in different lignin types [36], and serving as a rapid method for monitoring lignin decomplexation reactions [4,25,37,38]. Figure 3 and Figure 4 exhibit the FTIR spectra of the DL products obtained through different depolymerization times. The full-range Spectrum (Figure 3) shows that O-H stretching vibrations are evident between 3600 and 3100 cm^−1^, while C-H stretching vibrations are observed at 2961, 2918, 2873, and 2850 cm^−1^. Most of the signals in the spectrum appear below 1800 cm^−1^ (Figure 4). A signal at 1730 cm^−1^ is attributed to carbonyl (C=O) stretching vibrations. Peaks at 1590 and 1443 cm^−1^ could indicate the presence of C-C double bonds. The signal at 1113 cm^−1^, assignable to C-O stretching vibrations, originates from ether bonds. Prominent signals around 1015 cm^−1^ suggest C-O-H and C O-C stretching vibrations. C-H deformation vibrations at 833 cm^−1^ point to the presence of aromatic compounds that are present in DL as well as in the reference reaction [39].

Lignin depolymerization is widely acknowledged to be a process with inherently low selectivity, producing a broad array of products [40]. As a result, the characterization of the extracted compounds often reveals a diverse spectrum of chemical species. This complexity also poses challenges in the precise quantification of the individual compounds, which in turn complicates the determination of critical reaction parameters such as yield and selectivity toward specific target molecules. The heterogeneity of the product mixture makes it difficult to pinpoint the exact quantities and proportions of each compound, limiting our ability to fully optimize the depolymerization process. To gain a deeper understanding of the composition of the DL products, high-resolution mass spectrometry was employed for direct injection analysis. This technique allowed for the identification of the major aromatic compounds (Table 2). One of the primary motivations for utilizing reductive fractionation in this study was to avoid overoxidation and undesirable condensation reactions, which can occur during lignin depolymerization [5,10,41]. These side reactions often lead to the formation of recalcitrant, high-molecular-weight byproducts that are difficult to valorize. By applying a reductive approach, we were able to mitigate these issues and primarily obtained products rich in aryl ether and phenolic functional groups (Table 2).

Previous studies have demonstrated that in the late-stage catalytic conversion of lignin, the lignin structure undergoes targeted breakdown toward the end of the depolymerization process, often resulting in the formation of a higher yield of monomeric and dimeric compounds [5]. This stage typically involves catalytic strategies that suppress undesired re-polymerization and condensation reactions, which can otherwise limit the yield and functionality of the resulting lignin-based feedstock. In alignment with these findings, our approach resulted in the production of not only lignin monomers but also lignin dimers (Table 2). These compounds closely resemble those typically obtained from reductive catalytic fractionation (RCF), which is known to produce a low-molecular-weight, highly functional lignin oil [42,43]. The presence of such a product profile suggests that our process avoids excessive condensation, yielding a lignin oil that is both chemically versatile and amenable to further refinement.

Table 3 presents the relative intensity of phenolic products as a function of depolymerization time. It can be observed that both the intensity and the presence of certain aromatic compounds decrease as the reaction progresses, indicating that lignin dearomatization is time-dependent. This result is consistent with our previous work [4], which showed that dearomatization is influenced not only by time but also by the applied current.

**Figure 5:**
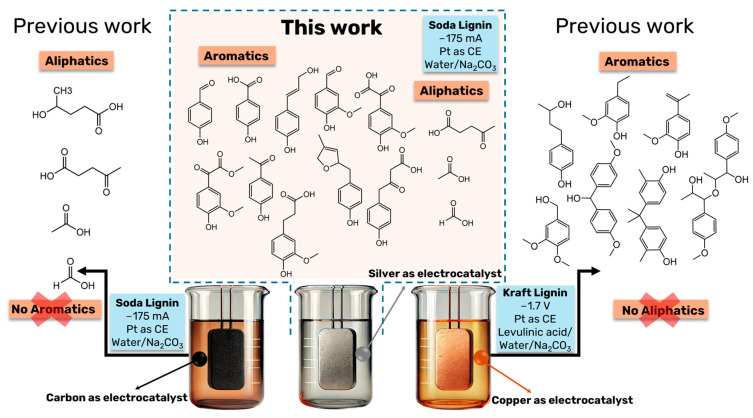
Comparison of DL products obtained in the current study with those from previous experiments, where soda lignin and kraft lignin were subjected to electrochemical reductive depolymerization under varying conditions [4,5]. This figure highlights the impact of using different electrocatalysts (silver, carbon, copper) and reaction parameters (current, time, and electrolyte composition) on the product distribution. The comparison illustrates how the choice of electrocatalyst significantly influences the selectivity toward aliphatic and aromatic compounds, with silver showing a broader range of products compared to previous studies using carbon and copper.

Figure 5 provides a comparison between the DL products obtained in this work to previous ones where soda lignin, as well as kraft lignin, has been also depolymerized under electrochemical reductive conditions.

In our pioneering work on the electrochemical depolymerization of lignin under reductive conditions, copper was employed as the electrocatalyst, with kraft lignin as the substrate, and a solvent system composed of levulinic acid and water [5]. The electrolyte, Na_2_CO_3_, was the same as in the present study. Unlike the current work, which utilizes chronopotentiometry, the previous study used chronoamperometry at a fixed potential of −1.7 V for 20 h. For comparison, the resulting current density in that study was 9 mA cm^−2^, considerably lower than the −175 mA cm^−2^ employed here. The earlier study primarily identified aryl ether and phenolic groups in the depolymerized lignin (DL) products, with no aliphatic compounds detected (Figure 5). In contrast, in another earlier work, using carbon as the electrocatalyst and soda lignin as the substrate, no aromatic compounds were observed after 20 h of depolymerization at −175 mA in a water/sodium carbonate solvent system [4]. Instead, the reaction was highly selective toward four major aliphatic products: sodium levulinate, sodium 4-hydroxyvalerate, sodium formate, and sodium acetate. Except for sodium 4-hydroxyvalerate, all these aliphatic compounds have also been identified in the present work. However, the presence of aromatic compounds in the present work suggests reduced selectivity when silver was employed as the electrocatalyst.

The observed differences in product distribution when copper, carbon, or silver are employed as electrocatalysts could be attributed to the distinct catalytic properties and surface interactions of these materials. Copper, known for its strong catalytic activity in reductive processes, tends to favor the cleavage of aryl ether bonds, leading to a higher yield of phenolic compounds in the depolymerized lignin [5]. Although silver is often associated with high selectivity in catalytic reactions [44,45] due to its unique electronic properties, it seems to facilitate both aromatic and aliphatic product formation in this case. This could be attributed to silver’s affinity for oxygen-containing functional groups [46], which would favor the simultaneous cleavage of different bond types within lignin. Thus, the choice of electrocatalyst significantly impacts product distribution, with silver showing a broader range of products, including both aromatic and aliphatic compounds.

## 4. Conclusions

This study successfully demonstrates a silver-catalyzed electrochemical method for selective depolymerization and partial dearomatization of soda lignin under ambient, mild conditions, representing a significant advance in lignin valorization. Utilizing a sodium carbonate-based solvent system, the process achieved conversion of lignin into valuable aliphatic and aromatic compounds, as evidenced by yields up to 21.8 wt% of depolymerization products over a reaction time of 10 h. NMR and HRMS analyses identified sodium levulinate, sodium acetate, and sodium formate as principal aliphatic products, while various phenolic compounds were identified in the aromatic fraction via Direct infusion (DI) ESI-HRMS. The use of silver as an electrocatalyst enabled a broader range of product formation than previous studies with copper and carbon, though with lower selectivity toward aliphatic products. This process not only optimizes lignin depolymerization under mild conditions but also highlights silver’s potential to drive sustainable bio-based chemical production, contributing to circular economy goals by reducing reliance on fossil-based resources. Further optimizations in catalyst design and reaction parameters could refine the selectivity and yield, paving the way for scalable applications in sustainable bio-refining.

## Figures and Tables

**Table 1:** Yields of depolymerization products (DL) relative to the mass of products obtained after ethanol extraction, compared to the initial mass of soda lignin. All reactions were carried out at room temperature and ambient pressure with a constant current of −175 mA. The yield from soda lignin corresponds to the ethanol-soluble fraction of untreated soda lignin (i.e., without electrocatalysis). The yield at 0 h refers to the ethanol-soluble fraction of soda lignin dissolved in a water/sodium carbonate solution and left for 20 h without undergoing electrocatalysis.

Duration	Soda Lignin	0 h	5 h	10 h	15 h	20 h
Yield [wt%]	7	6	13.4	21.8	18.33	18.35

**Table 2:** Main phenolic products of depolymerization identified by direct infusion (DI) ESI-HRMS.

***m*/*z***	121.0292	137.0272	149.0605	151.0400	165.0193
Chemical Structure	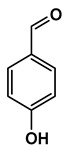	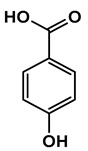	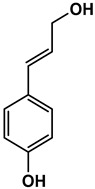	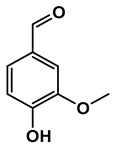	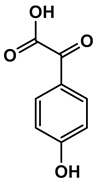
	4-hydroxybenzaldehyde	4-hydroxybenzoic acid	(E)-4-(3-hydroxyprop-1-en-1-yl)phenol	4-hydroxy-3-methoxybenzaldehyde	2-(4-hydroxy-3-methoxyphenyl)-2-oxoacetic acid
***m***/***z***	165.0552	189.0915	193.0504	195.0297	195.0661
Chemical Structure	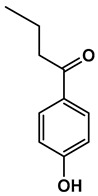	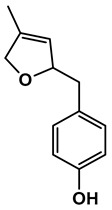	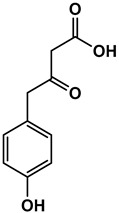	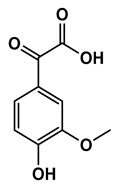	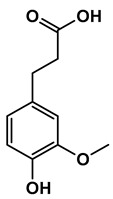
	1-(4-hydroxyphenyl)butan-1-one	4-((4-methyl-2,5-dihydrofuran-2-yl)methyl)phenol	4-(4-hydroxyphenyl)-3-oxobutanoic acid	2-(4-hydroxy-3-methoxyphenyl)-2-oxoacetic acid	3-(4-hydroxy-3-methoxyphenyl)propanoic acid

**Table 3:** Relative intensity of phenolic products as a function of depolymerization time.

***m*/*z***	0 h	5 h	10 h	15 h	20 h
121.0292					
137.0272					
149.0605					
151.0400					
165.0193					
165.0552					
189.0915					
193.0504					
195.0297					
195.0661		--	--	--	--
Intensity	maximum	medium	low	--	--

## Data Availability

The original contributions presented in the study are included in the article; further inquiries can be directed to the corresponding author.

## Supplementary Materials

The following supporting information can be downloaded at: https://www.mdpi.com/article/10.3390/polym16233325/s1, Figure S1: Cyclic voltammogram assesses the stability of the silver electrode in lignin solution during the electrochemical reaction.
