# Supplementary material for: Silver-Catalyzed Aqueous Electrochemical Valorization of Soda Lignin into Aliphatics and Phenolics
Source: Polymers (Basel). 2024 Nov 27;16(23):3325. doi: 10.3390/polym16233325 (PMC11644402; doi:10.3390/polym16233325)
Supplement: Supplementary file 1 [file polymers-16-03325-s001.zip › polymers-3293484-supplementary.pdf]

Electronic Supplementary Information (ESI)

Silver-Catalyzed Aqueous Electrochemical Valorization of Soda Lignin into Aliphatics and Phenolics

Lucie Lindenbeck [1], Silas Brand [1], Franka Stallmann [1], Vanessa Barra [1], Marcella Frauscher [2], Björn B. Beele [1], Adam Slabon [1,3]*, and Bruno V. Manzolli Rodrigues [1]*

[1] Faculty of Mathematics and Natural Sciences: Chair of Inorganic Chemistry, Faculty of Mathematics and Natural Sciences, University of Wuppertal, Gaußstraße 20, 42119 Wuppertal, Germany

[2] AC2T Research GmbH, Viktor-Kaplan-Straße 2, 2700 Wiener Neustadt, Austria

[3] Wuppertal Center for Smart Materials & Systems, University of Wuppertal, 42119 Wuppertal, Germany

*Corresponding author e-mail: slabon@uni-wuppertal.de; manzolli@uni-wuppertal.de

Stability of the Silver Electrode.. 2

Stability of the Silver Electrode

To determine the stability of the electrode material, a cyclic voltammetry (CV) experiment was conducted in a lignin solution (3 g·L^{-1}). The potential range was set to −1.5 to −2.5 V vs Ag/AgCl (satd. KCl), and no peaks corresponding to reduction or oxidation were observed. This indicates that the electrode material exhibited high stability under these reaction conditions.

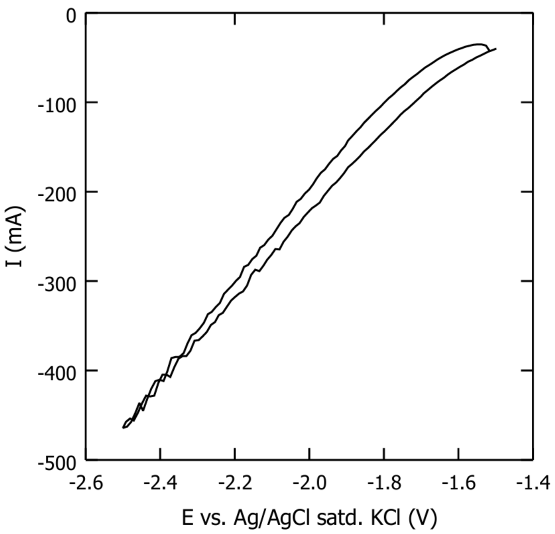

Figure S 1. Cyclic voltammogram assess the stability of the silver electrode in lignin solution during the electrochemical reaction.
